# A Case of Co-occurrence of COVID-19 and Group A Streptococcal Pharyngitis

**DOI:** 10.7759/cureus.14729

**Published:** 2021-04-28

**Authors:** Kok Hoe Chan, Sindhusha Veeraballi, Eyad Ahmed, Ramy Yakobi, Jihad Slim

**Affiliations:** 1 Internal Medicine, Saint Michael's Medical Center, Newark, USA; 2 Emergency Medicine, Saint Michael's Medical Center, Newark, USA; 3 Infectious Diseases, Saint Michael's Medical Center, Newark, USA

**Keywords:** group a streptococcus, covid-19, sars-cov-2, acute pharyngitis, sore throat

## Abstract

Coronavirus disease 2019 (COVID-19) has swept the world with over hundred million of cases and millions of deaths. Upper respiratory tract symptoms including acute pharyngitis are the common symptoms of COVID-19, with a reported incidence of about 5%-17.4%. Group A Streptococcus (GAS) pharyngitis is a common cause of bacterial pharyngitis, with highest incidence between age 5 and 15, and it can still occur in adults with peak incidence at age 40. Herein, we report a case of co-occurrence of GAS and COVID-19 in a middle-aged man who presented with fever, sore throat, cough, and runny nose. To the best of our knowledge, we are the first to report this unique co-occurrence. Our case report aimed to raise the awareness among physician particularly in ambulatory and emergency department of not to have a singular focus on COVID-19 and forget to screen patient with acute pharyngitis for GAS.

## Introduction

Coronavirus disease 2019 (COVID-19) is an ongoing global pandemic caused by severe acute respiratory syndrome coronavirus 2 (SARS CoV-2) [1]. Since its outbreak in December 2019, it has been rapidly progressing all over the world. As of April 4th, 2021, 130 million COVID-19 cases were confirmed globally with 2.8 million deaths [1].

COVID-19 has a multifaceted presentation with the majority of patients presenting with upper respiratory tract symptoms. Acute pharyngitis is not an uncommon symptom of COVID-19 and has been reported in about 5%-17.4% of patients with COVID-19 [2-4]. On the other hand, not all acute pharyngitis are related to COVID-19 even during this COVID-19 pandemic. Herein, we report a case of group A Streptococcus (GAS) pharyngitis and COVID-19 coinfection in a 38-year-old patient who presented to the emergency department with sore throat and upper respiratory tract symptoms. We hope to raise the awareness among the physicians to not forget to screen for this common infection in patients with sore throat during this COVID-19 pandemic.

## Case presentation

A 38-year-old gentleman with no significant past medical history presented to the emergency department with intermittent low-grade fever, productive cough with yellowish sputum, running nose, and sore throat for three days duration. He also complained of loss of taste, loss of smell, and poor oral intake. Otherwise, he denied chest pain, shortness of breath, chills, rigors, night sweats, headache, dizziness, abdominal pain, change in bowel movement, and urinary symptoms. He also denied having any sick contacts or known contact with patients with COVID-19.

Initial vital signs showed temperature of 101.4 °F, blood pressure of 114/70 mmHg, pulse rate of 115 beats/min, respiratory rate of 20 breaths/min, and saturating at 98% on room air. Physical examination was significant for pharyngeal and tonsil edema and erythema with exudates. Neck examination revealed right anterior cervical lymphadenopathy measuring about 1 x 2 cm in size, mobile, tender without any overlying skin changes or drainage. Lungs examination was notable for bilateral crackles diffusely. Otherwise, no rashes or petechiae were noted. Cardiovascular examination was unremarkable. 

**Figure 1:**
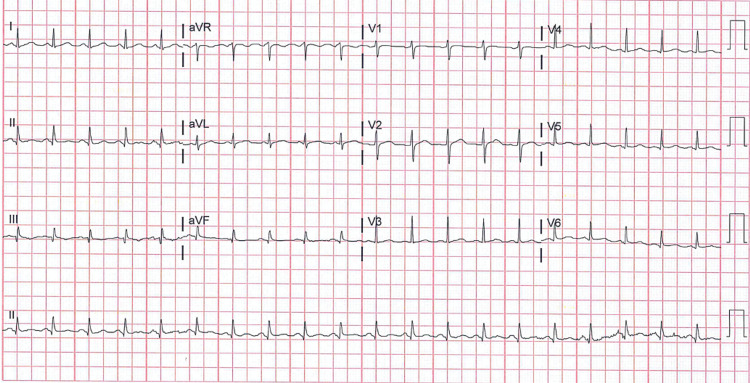
Electrocardiogram revealed sinus tachycardia with heart rate of 120 with no significant ST and T wave changes.

His complete blood cell count was unremarkable with hemoglobin 15 g/dl (normal: 13.5-17.5), hematocrit 43% (normal: 41-50), white cell counts 6,900 cells/microliter (normal: 4,500-11,000), and platelets 193,000 cells/microliter (normal: 150,000-450,000). Lactic acid was 4.5 mmol/l (normal: 0-2) and D-dimer 303 ng/ml (normal: 0-500). Electrocardiogram revealed sinus tachycardia with heart rate of 120 with no significant ST and T wave changes (Figure 1).

**Figure 2:**
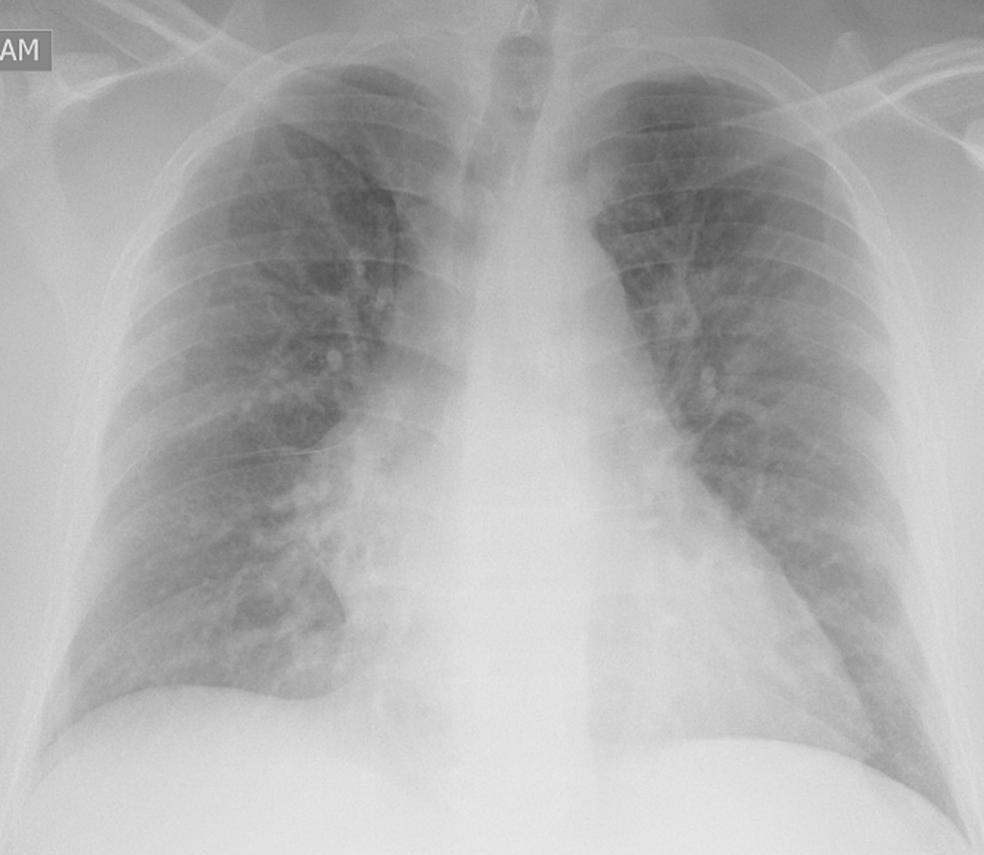
Chest X-ray revealed diffuse bilateral infiltrates, consistent of COVID-19 pictures

His chest X-ray revealed diffuse bilateral infiltrates, consistent of COVID-19 pictures (Figure 2). 

In light of the suspicion of GAS pharyngitis and COVID-19, SARS-CoV-2 reverse transcription polymerase chain reaction and rapid Streptococcus A test were ordered, and both came back positive. He was treated with a dose of ceftriaxone and azithromycin in the emergency department and was sent home with azithromycin 250 mg for four days with instructions to maintain social distancing, isolation, mask wearing, and hand washing.

## Discussion

Acute pharyngitis is one of the common conditions encountered in both emergency department and ambulatory practice. Acute pharyngitis commonly occurs in children and adolescents but do occur in adults with peak incidence at the age of 40 [5]. Acute pharyngitis can be classified into infectious, which is usually caused by bacteria or viruses, and noninfectious commonly due to allergic rhinitis, gastroesophageal reflux, or chemical irritation. Most of the acute infectious pharyngitis caused by respiratory virus are self-limited. However, the symptoms of respiratory virus pharyngitis also broadly overlap with GAS leading to often misdiagnosis.

SARS-CoV-2 is one of the leading causes of acute viral pharyngitis during this pandemic. With the emerging of this pandemic, there is a risk of colluding judgment with more emphasis on COVID-19 symptoms, especially with the overlapping signs and symptoms of COVID-19 with many other bacterial and viral infections potentiating the risk of missing common pathogens. GAS pharyngitis is one of the most common bacterial pharyngitis in adults with reported incidence of 5%-15% in developed countries [6-8].

Diagnosing GAS pharyngitis is paramount to prevent the complications associated with untreated conditions. The Centor score, a four-point scoring system that consists of fever, anterior cervical lymphadenopathy, tonsillar exudates, and absence of cough, has been validated and used in risk stratification for patients suspected to have GAS pharyngitis [9]. Our patient scored 3 in the Centor scoring system, although he presented with cough and highly suspicious of COVID-19, but the physical examination of tonsillar erythema with exudates and anterior cervical lymphadenopathy prompted us to screen for GAS.

GAS pharyngitis is a treatable condition. The antibiotic of choice is mainly penicillin, and in patients with penicillin allergy, cephalosporin or macrolides can be used [10-12]. The treatment of GAS is paramount to avoid complications. Untreated GAS can lead to varieties of complications, which are broadly divided into suppurative and non-suppurative [13]. Non-suppurative complications include acute rheumatic fever, acute glomerulonephritis, post-Streptococcal reactive arthritis, and Streptococcal toxic shock syndrome. On the other hand, suppurative complications include deep tissue infections with tonsillo-pharyngeal abscess, jugular vein thrombophlebitis, otitis media, sinusitis, and necrotizing fasciitis [13]. 

In our case, we presented a middle-aged gentleman with classic COVID-19 symptoms along with symptoms of GAS pharyngitis. When there is more emphasis on COVID-19, there is a greater chance of missing the symptoms of common pathogens especially in the emergency setting. With this case, we hope to raise the awareness among the physicians as to not miss the most common and potentially lethal acute pharyngitis in the emergency setting during the COVID-19 pandemic.

## Conclusions

In conclusion, COVID-19 has overwhelmed the healthcare system. Our case highlights the need to keep an open mind for diagnosing other infectious etiologies that may have overlapping symptoms with SARS-CoV-2 symptomatology. The co-occurrence of COVID-19 and GAS may be more common than what we thought. There is a need to widen our vision in order to provide quality care to the patients. We recommend testing for common pathogens and avoid the singular focus on COVID-19.

## References

[1] (2021). World Health Organization. Weekly epidemiological update on COVID-19 - 6 April 2021. https://www.who.int/publications/m/item/weekly-epidemiological-update-on-covid-19---6-april-2021.

[2] Chen N, Zhou M, Dong X (2020). Epidemiological and clinical characteristics of 99 cases of 2019 novel coronavirus pneumonia in Wuhan, China: a descriptive study. Lancet.

[3] Guan WJ, Ni ZY, Hu Y (2020). Clinical characteristics of coronavirus disease 2019 in China. N Engl J Med.

[4] Wang D, Hu B, Hu C (2020). Clinical characteristics of 138 hospitalized patients with 2019 novel coronavirus-infected pneumonia in Wuhan, China. JAMA.

[5] André M, Odenholt I, Schwan A (2002). Upper respiratory tract infections in general practice: diagnosis, antibiotic prescribing, duration of symptoms and use of diagnostic tests. Scand J Infect Dis.

[6] Snow V, Mottur-Pilson C, Cooper RJ (2001). Principles of appropriate antibiotic use for acute pharyngitis in adults. Ann Intern Med.

[7] Centor RM, Atkinson TP, Ratliff AE (2015). The clinical presentation of Fusobacterium-positive and streptococcal-positive pharyngitis in a university health clinic: a cross-sectional study. Ann Intern Med.

[8] Shulman ST, Bisno AL, Clegg HW (2012). Clinical practice guideline for the diagnosis and management of group A streptococcal pharyngitis: 2012 update by the Infectious Diseases Society of America. Clin Infect Dis.

[9] Centor RM, Witherspoon JM, Dalton HP, Brody CE, Link K (1981). The diagnosis of strep throat in adults in the emergency room. Med Decis Making.

[10] Skoog Ståhlgren G, Tyrstrup M, Edlund C (2019). Penicillin V four times daily for five days versus three times daily for 10 days in patients with pharyngotonsillitis caused by group A streptococci: randomised controlled, open label, non-inferiority study. BMJ.

[11] Tack KJ, Hedrick JA, Rothstein E, Nemeth MA, Keyserling C, Pichichero ME (1997). A study of 5-day cefdinir treatment for streptococcal pharyngitis in children. Cefdinir Pediatric Pharyngitis Study Group. Arch Pediatr Adolesc Med.

[12] Casey JR, Pichichero ME (2005). Higher dosages of azithromycin are more effective in treatment of group A streptococcal tonsillopharyngitis. Clin Infect Dis.

[13] Ashurst JV, Edgerley-Gibb L (2021). Streptococcal Pharyngitis. http://www.ncbi.nlm.nih.gov/books/NBK525997/.

